# An effective molecular approach for assessing cereal aphid-parasitoid-endosymbiont networks

**DOI:** 10.1038/s41598-017-02226-w

**Published:** 2017-06-09

**Authors:** Zhengpei Ye, Ines M. G. Vollhardt, Susanne Girtler, Corinna Wallinger, Zeljko Tomanovic, Michael Traugott

**Affiliations:** 1 Mountain Agriculture Research Unit, Institute of Ecology, University of Innsbruck, Innsbruck, Austria; 2 Agroecology, Department of Crop Sciences, Georg-August-University Göttingen, Göttingen, Germany; 3 Institute of Zoology, Faculty of Biology, University of Belgrade, Belgrade, Serbia

## Abstract

Molecular approaches are increasingly being used to analyse host-parasitoid food webs as they overcome several hurdles inherent to conventional approaches. However, such studies have focused primarily on the detection and identification of aphids and their aphidiid primary parasitoids, largely ignoring primary parasitoid-hyperparasitoid interactions or limiting these to a few common species within a small geographical area. Furthermore, the detection of bacterial secondary endosymbionts has not been considered in such assays despite the fact that endosymbionts may alter aphid-parasitoid interactions, as they can confer protection against parasitoids. Here we present a novel two-step multiplex PCR (MP-PCR) protocol to assess cereal aphid-primary parasitoid-hyperparasitoid-endosymbiont interactions. The first step of the assay allows detection of parasitoid DNA at a general level (24 primary and 16 hyperparasitoid species) as well as the species-specific detection of endosymbionts (3 species) and cereal aphids (3 species). The second step of the MP-PCR assay targets seven primary and six hyperparasitoid species that commonly occur in Central Europe. Additional parasitoid species not covered by the second-step of the assay can be identified via sequencing 16S rRNA amplicons generated in the first step of the assay. The approach presented here provides an efficient, highly sensitive, and cost-effective (~consumable costs of 1.3 € per sample) tool for assessing cereal aphid-parasitoid-endosymbiont interactions.

## Introduction

Host-parasitoid food webs are among the most studied terrestrial feeding networks and they have been investigated as models to address important questions in network ecology: for example, these networks have been investigated to better understand robustness and restoration of ecological networks^1^, apparent competition^2, 3^, bottom-up effects on food web structure^4^ as well as effects of habitat modification^5^, the role of evolutionary processes on food webs^6^ and host specificity^7, 8^. Parasitoids also play an important role in biological control of pests, such as aphids, which feed on a variety of plants, including a number of economically important crops. Cereals are often heavily infested with aphids, and are credited with being the most important cereal pests in the last 30 years^9^. Hymenopteran parasitoids play an important role in cereal aphid suppression^10^. However, the biological control exerted by primary parasitoids can be disrupted by hyperparasitoids (secondary parasitoids)^11–13^. These hyperparasitoids attack the primary parasitoid either inside the living (“true” hyperparasitoids) or the mummified aphid (mummy parasitoids)^14, 15^. Furthermore, as originally demonstrated in pea aphids^16^, aphid-parasitoid interactions can be affected by vertically-transmitted facultative bacterial endosymbionts which can confer aphid resistance to parasitoids. Two protective bacterial species of this kind are known to date, including *Hamiltonella defensa* ^17, 18^ and *Regiella insecticola* ^19, 20^. Additionally, the aphid X-type symbiont (PAXS) can be found in aphids and increases the protective power of *H. defensa* at high temperatures^21^. These defensive facultative endosymbionts can also be transferred between aphid species through horizontal transmission and as such potentially confer resistance to parasitoids in different aphid species^20, 22^. Also, parasitoids can act as vectors for the horizontal transmission of bacterial endosymbionts between aphids^23^. However, little is known about the occurrence of these endosymbionts in aphid populations and the functional role they play for their hosts. The occurrence of bacterial endosymbionts in cereal aphids has been reported in the literature: *H. defensa* and *R. insecticola* have been found in *S. avenae* and *Metopolophium dirhodum*; and to date, the occurrence of facultative endosymbionts in *Rhopalosiphum padi* remains unknown^22, 24–26^. Interestingly, *H. defensa*-infected *S. avenae* seem to be less attractive hosts for parasitoids, although a defensive effect of facultative endosymbionts has not yet been observed in natural colonies of cereal aphids^22^.

Assessing interactions between hosts, primary parasitoids and hyperparasitoids as well as facultative endosymbionts is difficult by conventional approaches^27^, such as parasitoid rearing and host dissection. These methods usually do not allow the identification of linkages between hyperparasitoids and their specific primary parasitoid host species^28^. The same applies for detecting endosymbionts within hosts, the prerequisite to examine how facultative endosymbionts affect host-parasitoid interaction networks. DNA-based approaches have been shown to overcome these limitations as they allow the identification of species-specific links between primary parasitoids and hyperparasitoids^29–31^ and can also detect and identify the presence of specific facultative endosymbionts^25, 32^. These approaches are not affected by delayed parasitoid emergence, host and parasitoid mortality; they are capable of detecting and identifying multiple parasitoid species simultaneously^29–31, 33^. Currently, there are two kinds of molecular approaches used to detect and identify parasitoids and host endosymbionts: (i) diagnostic PCR and (ii) DNA sequence-based methods^34^. The latter has primarily been used to identify parasitoid DNA using universal primers followed by Sanger sequencing^35^ or next generation sequencing (NGS)^36^. However, Sanger sequencing is not ideal to assess aphid-parasitoid-endosymbiont interactions in aphids, as it is difficult to obtain high-quality DNA sequence data for multiple species in the same DNA extract, as is typically encountered in hyperparasitized and multiparasitized aphids^35^. The NGS approach can overcome this hurdle, but it becomes increasingly costly and time consuming when a large number of specimens need to be sequenced individually, as is typically required to establish quantitative food webs. In addition, primers uniquely designed to amplify parasitic hymentoptera (including parasitoids and hyperparasitoids) have not yet been described, which has two important implications for an NGS approach: (i) existing general barcoding primers would need to be evaluated for their coverage of the wide range of primary parasitoid and hyperparasitoids species and (ii) general barcoding primers will also amplify host DNA and can prohibit the detection of parasitoid DNA (especially in cases where only small amounts of parasitoid DNA are present such as in eggs and early instar larvae) as large amounts of host DNA will preferentially generate host sequences. Multiplex PCR (MP-PCR) can overcome these limitations for a defined set of parasitoid taxa as it allows the amplification of several targets in parallel within a single reaction^11, 29–31^. However, most MP-PCR approaches have been developed for specific aphid-parasitoid systems, and are focused on parasitoid species or genera that are locally or regionally important within a certain geographical area^29–31^. A universal MP-PCR system that targets multiple species of primary parasitoids and hyperparasitoids would enable a broader application of this approach. There is currently no molecular system which allows for the simultaneous detection and identification of aphid, primary parasitoid, hyperparasitoid, and facultative endosymbiont DNA. However, such a tool is critical for the examination of aphid-parasitoid-endosymbiont networks under field conditions^26, 37^, and an improved understanding of these networks may have implications for integrated pest management strategies for aphids.

To address this, the present study describes the development of a two-step MP-PCR approach to assess the occurrence of parasitoids and facultative endosymbionts in cereal aphid-primary parasitoid-hyperparasitoid communities of Central Europe. The first step in the MP-PCR approach allows for the general detection of parasitoids in cereal aphids and the species-specific identification of three facultative endosymbiont species. The second step of the MP-PCR approach enables species-level identification of the most common primary parasitoid and hyperparasitoid species. In addition, the present approach includes a DNA-sequence based method for species-level identification of less common parasitoid taxa which are not covered in the MP-PCR. To evaluate the utility of this approach, experiments to determine the sensitivity and specificity of the assay were conducted, and field-collected aphid samples were screened.

## Results

### Multiplex PCR assays

With the 18SMP assay, a 196 ± 4 bp (193, 203 bp) 18S fragment was amplified from all the 31 primary parasitoid and 16 hyperparasitoid species examined in this study (amplicon length is provided with mean ± SD, parenthesis: minimum, maximum; Table 1). Additionally, species-specific amplicons ranging between 105 and 604 bp (Table 1) for each of the cereal aphids and their facultative endosymbionts were produced. The 16SMP assay included the same primer pairs for facultative endosymbionts and aphids, except for *R. padi* (478 bp amplicon; Table 1). Parasitoid DNA was targeted by three specific amplicons: (i) a 213 ± 2 bp (211, 216 bp) fragment for 26 aphidiid species, (ii) a 317/318 bp fragment for two *Dendrocerus* species, and (iii) a 155 ± 2 bp (152, 158 bp) amplicon for 14 other hyperparasitoid and five *Aphelinus* species (Table 1).

**Table 1:** Primers used in the first step (18SMP and 16SMP) and second step (PriMP and HypMP) multiplex PCR assays including assay names, primer targets, primer sequences (forward followed by reverse primers), the expected product size, concentration of each primer (Conc.) in the PCR, the gene of the primers are based on, and the detection limits expressed as DNA template copies per reaction.

Assay name	Targeted taxa	Primer names and sequences (5′-3′)	Product size (bp)	Conc. (µM)	Gene	Detection limit (templates)
18SMP	Parasitoid-group	ParG-S452: AACYAGARTTCCRACCAGAGATGG	196 ± 4 (193, 203)	1	18S	/
		ParG-A435: CATGGTAGGCRYAGAACCTACCA				
	*Rhopalosiphum padi*	Rho-pad-S440: TAATAATATAAAATTAAACCAAATTCCATTA	136	0.2	COI	750
		Rho-pad-A442: TGATGTATTTAAATTACGATCAGTAAGAAG				
	*Sitobion avenae*	Sit-ave-S433: TCATCACTTAGAATTCTTATTCGTCTT	259	0.08	COI	750
		Sit-ave-A437: AGAAACTACAGATTATTATTATTAATGATGGT				
	*Metopolophium dirhodum*	Met-dir-S436: CCTTTATCAAATAACATTGCACATAAC	105	0.22	COI	750
		Met-dir-A440: AATAAAGTTAATTGCTCCTAAAATTGAG				
	*Hamiltonella defensa*	Ham-def-S441: TTTGGAGGTTGCGGTCTTG	233	0.02	16S	150
		Ham-def-A443: CCATGCAGCACCTGTCTCAC				
	*Regiella insecticola*	Reg-ins-S443: AAGTTGCCTTCGGGAGCC	426	0.022	16S	150
		Reg-ins-A446: AAGCTACCTACTTCTTTTGCCG				
	Pea aphid X-type symbiont (PAXS)	PAXS-S447: ACTGTGGCTTGCGGAGCA	604	0.038	16S	150
		PAXS-A450: TAAGCTACCTACTTCTTTTGCAAAA				
16SMP	Aphidiinae	AphG-S458: WATAATYTTAAGTCWAATCTGCC	213 ± 2 (211, 216)	1	16S	15
		ParG-A462: AARTTCTAWAGGGTCTTMTCGTCT				
	*Aphelinus*-Hyperparasitoid	HypG-S460: TAAYTGTACWAAGGTAGCATAATCAWTT	155 ± 2 (152, 158)	0.8	16S	15
		ParG-A462: AARTTCTAWAGGGTCTTMTCGTCT				
	*Dendrocerus* spp.	DenG-S461: CTAAGGTAGCATAATAATTAGTTTATTAATTGT	317/318	0.2	16S	15
		DenG-A461: GCTGTTATCCCTAAAGTAATTTAATCA				
	*R. padi*	Rho-pad-S437: CCTTAGAATCTTAATTCGAYTAGAACTAAGT	478	0.14	COI	7500
		Rho-pad-A458: TGTAATTAAAATTGATCAAGGGAATAAT				
	*S. avenae*	Sit-ave-S433/Sit-ave-A437	259	0.08	COI	7500
	*M. dirhodum*	Met-dir-S436/Met-dir-A440	105	0.12	COI	7500
	*H. defensa*	Ham-def-S441/Ham-def-A443	233	0.02	16S	750
	*R. insecticola*	Reg-ins-S443/Reg-ins-A446	426	0.022	16S	750
	PAXS	PAXS-S447/PAXS-A450	604	0.038	16S	750
PriMP	*Aphidius ervi*	Aph-erv-S817: TCATGCTTTTGTAATAATTTTYTTTATG	324	0.4	COI	75
		Aph-erv-A816: ATAGTACTAATAAAATTAATAGCTCCYATRATAGAG				
	*Aphidius avenae*	Aph-pic-S820: CGAATAGAATTAAGARTTACTGGTACTTTT	605	1	COI	75
		Aph-pic-A820: CCAAAAAATCAAAATAAATGTTGATAC				
	*Aphidius uzbekistanicus*	Aph-uzb-S821: TTTATTTTTGGTATATGATCTGGAGTC	290	0.2	COI	75
		Aph-uzb-A821: CCTCTAATTAATAATAAAATTAAAGATGGAAC				
	*Ephedrus plagiator*	Eph-pla-S823: TTAGGTCATAGAGGAATATCAGTTGATA	134	0.4	COI	75
		Eph-pla-A824: TGATCYTTAACTATTCCTAAAGAATTTATTG				
	*Praon gallicum*	Pra-gal-S824: GATACCTGGTAGATTAATTGGAAGG	482	0.4	COI	75
		Pra-gal-A826: CTAAAACTGGTAAAGATAACAATAAMAATAA				
	*Praon volucre*	Pra-vol-S828: TGGAATATGAGCAGGAATAGTAGGRT	371	0.2	COI	75
		Pra-vol-A829: CTAAATCTACAGARATTCCYCTATGTCTAG				
	*Aphidius* spp.	AphG-S818: GAGAAGACCCTTTAGAATTTTATAWTAAWT	180/181	2.4	16S	150
		AphG-A818: CCCTAAGGTAATTTATTTTAAWATWCTAAAAA				
HypMP	*Aphelinus abdominalis*	Aphe-abd-S802: TGGTTTTTTGATTATAATTTAAAATCTG	368	0.8	16S	1500
		Aphe-abd-A802: TTATTTCAAAATAATTTGGATAAAATAAATA				
	*Alloxysta victrix*	All-vic-S801: AACAAATTTTTTTATTYTAAATAATACTTATAAG	101	0.4	16S	750
		All-vic-A801: TTAYGCTGTTATCCCTAAGGTAACTAG				
	*Asaphes suspensus*	Asa-sus-S808: AAAGACGAGAAGACCCTATAGAATTTA	138	1.8	16S	15000
		Asa-sus-A806: RAWTRATGTTAATTTATTAAAAAAATTTATTAAT				
	*Asaphes vulgaris*	Asa-vul-S809: ATTATTAAAAGACGAGAAGACCCTG	173	1	16S	1500
		Asa-vul-A807: TCATAATTTATGGATAAAAAATTTATTAATATATA				
	*Coruna clavata*	Cor-cla-S811: ACAAAGGTAGCATAATCAATTGTCTTA	255	0.8	16S	15000
		Cor-cla-A809: TATAAATTTATTTAAAAAATTTATTAAATTTTTAC				
	*Dendrocerus carpenteri*	Den-car-S814: TTATAGGATCAATTAACTTTTTRTCWACTCTAC	205	0.1	COI	150
		Den-car-A812: CCTCCWCCRCATGGGTCAAAG				
	*Phaenoglyphis villosa*	Pha-vil-S816: TCTTAATAATATAAGATACTGATTATTATTACCAG	306	0.2	COI	150
		Pha-vil-A815: GGTAAAGATAATAATAATAAAATTGTTGTTAGG				

**Figure 1:**
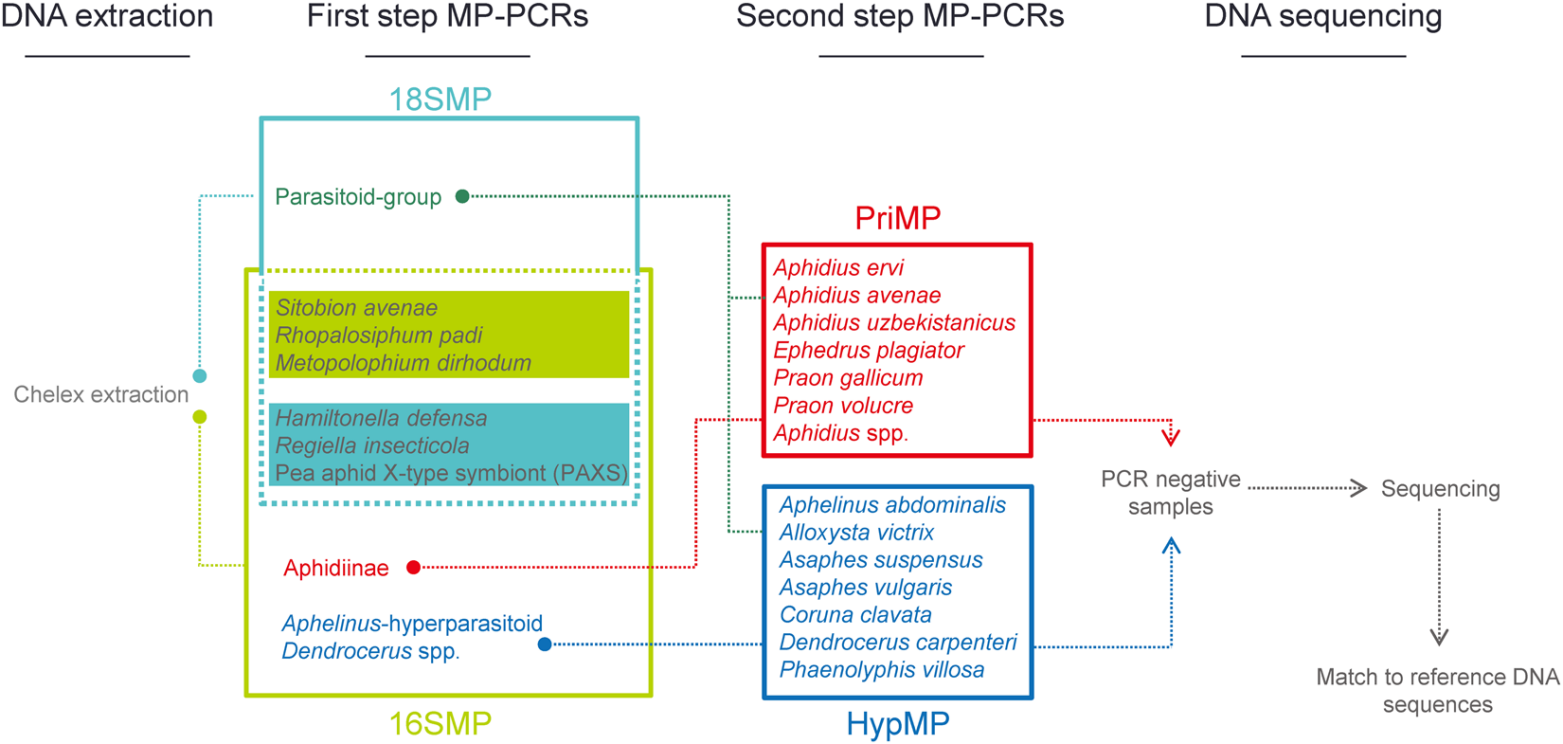
The two-step molecular approach for detecting and identifying three cereal aphid species (light green background), their primary parasitoids and hyperparasitoids as well as three facultative bacterial endosymbiont species (dark green background), including two multiplex PCR assays for each step and DNA sequencing.

For the identification of parasitoid species, samples which were positive for the presence of parasitoid DNA in the first step of the MP-PCR assay were tested with the second step MP-PCR assays (Fig. 1). The PriMP assay targeting the six Aphidiinae *Aphidius ervi*, *Aphidius avenae*, *Aphidius uzbekistanicus*, *Ephedrus plagiator*, *Praon gallicum* and *Praon volucre*, produced species-specific amplicons ranging between 134 and 605 bp (Table 1). Additionally, a 180 bp/181 bp *Aphidius*-group specific amplicon was included in this MP-PCR (Table 1) which targeted all *Aphidius* species except for *A. avenae*. The HypMP assay amplified a 368 bp fragment specific for the primary parasitoid *Aphelinus abdominalis* and species-specific amplicons for the hyperparasitoids *Alloxysta victrix*, *Asaphes suspensus*, *Asaphes vulgaris*, *Coruna clavata*, *Dendrocerus carpenteri* and *Phaenoglyphis villosa*, (101–306 bp; Table 1). All samples positive for parasitoid DNA with the 18SMP were tested with both of the second step MP-PCR assays to discriminate between primary parasitoids and hyperparasitoids, and detect species-specific trophic links between parasitoids and hyperparasitoids in parasitized aphids. In the 16SMP, the Aphidiinae-positive samples need to be subjected only to the PriMP, while the *Aphelinus*-hyperparasitoid- and *Dendrocerus*-positive have to be tested in the HypMP.

### Assay sensitivity and primer balancing

The sensitivity of the three parasitoid primers in the 16SMP was adjusted for 15 DNA templates (Table 1) to allow for the detection of parasitoid eggs in *S. avenae* immediately after oviposition (Table 2). The 18SMP allows amplification of parasitoid DNA as early as three days after oviposition (Table 2). For the PriMP, 75 DNA templates are sufficient to generate amplicons for each of the six primary parasitoid species, whereas 150 DNA templates of *A*. *rhopalosiphi* are required for a PCR product generated by the *Aphidius* group-specific primer pair (Table 1). In the HypMP, the sensitivities of the COI fragment-based detection of *D. carpenteri* and *P. villosa* is 150 DNA templates, with a considerably lower sensitivity for those species targeted by 16S-based primers: 750 DNA templates are needed for the *A. victrix* primer pair compared to 1,500 and 15,000 copies to produce PCR products for *A. abdominalis* and *A. vulgaris* as well as *A. suspensus* and *C. clavata*, respectively (Table 1).

**Table 2:** Number of positive parasitoid DNA detection in aphids collected at different time points post-parasitism with the first step MP-PCR assays (18SMP and 16SMP).

Assay name	Time points post-parasitism (# individuals tested)					
	1 h (n = 10)	1d (n = 10)	2d (n = 10)	3d (n = 10)	5d (n = 10)	7d (n = 8)
18SMP	0	0	0	4	5	5
16SMP	10	10	10	10	10	8

The number of individuals tested per time point for each assay is denoted in parentheses.

With both versions of the first step MP-PCR assays DNA of *H. defensa* could be detected in DNA extracts (n = 5) derived from *H. defensa*-infected “empty mummies”. The same was true for all 10 *H. defensa*-infected “full mummies”. None of the uninfected mummies tested positive for endosymbiont DNA. For the three facultative endosymbiont primer pairs a minimum of 150 and 750 DNA templates is needed to amplify species-specific PCR products with 18SMP and 16SMP, respectively (Table 1). The detection sensitivity of the aphid primers employed in the 18SMP and the 16SMP are 750 and 7,500 DNA templates, respectively (Table 1).

### Assay specificity

All newly developed MP-PCR assays are specific to their targeted taxa, comprising 47 parasitoid species, and the three facultative endosymbionts. All targeted taxa provide amplicons of the expected size and no cross-amplification was observed. There are only two exceptions in the 16SMP but these do not conflict with the detection of aphid and parasitoid DNA (details see Supplementary Appendix S1).

### Field sample tests

The newly-developed multiplex assays were applied to field-collected aphids (n = 42) and aphid mummies (n = 48) to validate their utility for the detection and identification of parasitoid, hyperparasitoid, and endosymbiont communities in nature. Thirteen aphids and 43 mummies scored positive for parasitoid DNA in the 18SMP, while 15 aphids and 48 mummies scored positive for parasitoid DNA in the 16SMP assay (Table 3). All diagnostic amplicons were confirmed as the respective target taxon via DNA sequencing. The 46 Aphidiinae-positive and 39 *Aphelinus*-hyperparasitoids/*Dendrocerus* spp.-positive samples in the 16SMP were tested in the second step PriMP and HypMP assays. Most of the parasitoid species were identified in the second step MP-PCRs, except for 10 parasitoid-positive samples: these samples, including four Aphidiinae and six *Aphelinus* sp. individuals or hyperparasitoids, were identified via DNA barcoding. Eight out of these 10 amplicons were identified to one of each *A. avenae, P*. *gallicum*, *P*. *villosa* and five *A*. *suspensus* individuals, while the other two Aphidiinae-amplicons were identified as *Aphidius* spp. and *Praon* spp., respectively. In 29 and 33 samples DNA of the facultative endosymbiont *H*. *defensa* and *R*. *insecticola*, respectively, was detected by the 18SMP assay. With the 16SMP, 25 *H. defensa*- and 29 *R*. *insecticola*-positives were obtained (Table 3), whereas no PAXS was detected at all. A much larger set of field-collected samples has been analysed with the current methodology to address various ecological questions and will be presented elsewhere.

**Table 3:** Detection rates of parasitoid DNA and facultative endosymbionts in field-collected aphids and mummies with the first step MP-PCR assays (18SMP and the 16SMP).

Sample type	Number	Parasitoids		Hamiltonella defensa		Regiella insecticola	
		18SMP	16SMP	18SMP	16SMP	18SMP	16SMP
Living aphids	42	31.0%	35.7%	38.1%	35.7%	57.1%	57.1%
Mummies	48	90.0%	100%	27.1%	20.8%	18.8%	10.4%
Total	90	62.2%	70%	32.2%	27.8%	36.7%	32.2%

## Discussion

In this study we present a new two-step molecular approach which allows the species-specific examination of cereal aphid primary parasitoid-hyperparasitoid interactions between the most common European species in these communities. In parallel, our assays allow species-specific identification of cereal aphids, and screens for the presence of facultative endosymbionts. The use of species-specific aphid primers not only permits rapid, conclusive identification of the aphid species collected, but also serves as an internal positive PCR control. With this setup, false negative results can be eliminated, thus circumventing the need for re-testing those samples which are negative for parasitoid and/or facultative endosymbiont DNA, an aspect that becomes particularly important at low parasitism and endosymbiont rates. The advantage to the two-step approach is that only those aphids which provide a positive PCR result using the general parasitoid primers need to be processed with the second step MP-PCR in order to identify which species are present. This greatly reduces the number of samples that need to be processed, as parasitism levels in cereal aphids are typically below 25%^30, 38, 39^. For those parasitoid and hyperparasitoid species which are less common and are not specifically targeted in the multiplex, identification can be accomplished by sequencing the 16S amplicons generated in the first step 16S MP-PCR and comparing the sequences with published or publically-available sequences^40^. These new general primer pairs for primary and hyperparasitoids will also prove useful in future studies using DNA sequence-based approaches for parasitoid identification such as Sanger sequencing or NGS, thereby extending their utility and making them broadly applicable to aphid-parasitoid systems on different host plants and in different geographic locations.

Hyperparasitoids play an important role in structuring aphid-parasitoid networks, but their impact is often overlooked because methodological difficulties prevent the link between the primary parasitoid and its hyperparasitoid being established^28^. Top-down effects of hyperparasitoids, often unperceived, must be considered when assessing the biological control services provided by cereal aphid primary parasitoids^11–13, 41^. In cases where heavy hyperparasitism occurs, apparent competition of aphids mediated by primary parasitoids has not been observed^3^; however, apparent competition between primary parasitoids mediated by hyperparasitoids has been demonstrated^42, 43^. The present MP-PCR assays allow the detection of primary parasitoid-hyperparasitoid interactions at species-specific level. Since higher trophic levels can be more sensitive to ecosystem disturbances such as land-use changes^44^, it is important to assess hyperparasitism and its prevalence in aphid-parasitoid food webs in different habitat^38, 45^. However, analysis of primary parasitoid-hyperparasitoid linkages is often fraught with difficulties: morphological approaches allow identification of primary parasitoids at the genus level (based on the shapes and colours of aphid mummies^46, 47^). Although it may be possible to link a hyperparasitoid species with the aphid species it emerges from, species-level identification of the primary parasitoid (i.e., the host of the hyperparasitoid) is lost^4, 45^. Only a few studies have employed molecular approaches to examine primary parasitoid-hyperparasitoid interactions at the species level^11, 29, 30, 39^. The present molecular approach significantly extends the species spectrum, as it allows the detection and identification of 24 primary parasitoid and 16 hyperparasitoid species of three cereal aphid species, which represent the predominant members of cereal aphid parasitoid guilds in Europe. Derocles, *et al*.^35^ employed a molecular approach to detect and identify parasitoids within the subfamily Aphidiinae based on DNA sequencing; however, their study did not include detection and identification of hyperparasitoids or *Aphelinus* primary parasitoids. In fact, to date only a single species of *Aphelinus* has been considered in molecular assays^29^. With the present molecular approach, all common members of this genus can be examined. Furthermore, according to the generality test with non-cereal aphid parasitoids, the 18SMP generates parasitoid amplicons across all 15 families, whereas the 16SMP generates Aphidiinae-amplicons from two genera of Braconidae and *Aphelinus*-hyperparasitoid amplicons across eight out of 15 families (see Supplementary Table S1). Therefore, the assays provided in the present study also have the potential to be adjusted and used in host-parasitoid interaction studies outside of Central Europe. The present tool is also cost-effective: the consumable costs (reagents and plastics) for the Chelex-based DNA extraction are approximately 0.23 € per sample. One MP-PCR reaction and its product visualization using the automatic capillary electrophoresis system QIAxcel (Qiagen) is approximately 1.07 € (again, both reagents and plastics). However, molecular methods may overestimate the real biocontrol efficiency of parasitoids on their aphid hosts, as they do not indicate whether parasitoid eggs and larvae survive within the host or if they are killed by host defence mechanisms, which has been discussed earlier^34, 48, 49^.

The molecular approach established here can also be used to screen simultaneously for three facultative endosymbiont species. It therefore saves a significant amount of time and money compared to already existing singleplex PCR assays^32^, and makes this a very promising tool in field studies - even at large scales. The present knowledge on facultative endosymbionts was primarily obtained from laboratory experiments^16^. So far, only a few field studies have investigated the role and function of facultative endosymbionts in aphids^22, 26, 32, 50^. Furthermore, the investigation of interactions between parasitoids and aphid defensive facultative endosymbionts are restricted to a few selected parasitoid and aphid species^26^, such as the pea aphid^16^. Accordingly, the role of different facultative endosymbionts in other aphid species remains unclear, although recent experimental work demonstrated behavioural effects of facultative endosymbionts on parasitoids^22^. The present MP-PCR approach now opens up a new avenue for field studies on endosymbiont-parasitoid interactions and paves the way to address questions that were previously unexplored in this field. It has been shown that the protective function of *H. defensa* depends on the presence of specific bacteriophages called APSEs (*A. pisum* secondary endosymbiont), which encode different toxins^17, 51^. In addition, the resistant strain of *R. insecticola* has been show to encode a pathogenicity pathway^52^. However, there is also some evidence that the resistance conferred by *H. defensa* may not depend on APSEs^53^. Nevertheless, it would be desirable for future work to design specific primers that target different strains within particular species of facultative endosymbionts. For example, the establishment of a strain-specific MP-PCR as an optional new second step MP-PCR assay could be useful in future studies.

In terms of assay sensitivity, the 16SMP allowed the detection of a single parasitoid egg within 1 h post-oviposition in the aphid host, and therefore exhibits a higher detection sensitivity than the 18SMP, which detected parasitoid DNA within an aphid approximately three days after observed oviposition. The sensitivity of the 16SMP was similar to the singleplex diagnostic PCR assay established by Traugott and Symondson^31^, whereas in other studies, parasitoid detection was not possible until 24 h after oviposition^35, 54^. The comparably low sensitivity of the 18SMP might be caused by the targeted gene. A single egg has only one nucleus but several mitochondria. Therefore, mitochondrial genes such as 16S and COI are present in much higher copy numbers than the nuclear 18S gene, likely leading to the higher sensitivity of primers targeting mitochondrial genes compared to the nuclear 18S region^55^. The sensitivity of the second step MP-PCR assays for parasitoid identification was generally lower than the 16SMP. However, only a low number of samples which tested positive for parasitoids in the first step MP-PCR failed to produce a species-specific band in the second step MP-PCR, indicating that the level of sensitivity was generally adequate for the detection and identification of aphid parasitoids and hyperparasitoids. However, when samples remain unidentified after the second step MP-PCR, they can still be identified via DNA sequencing of the first-step MP amplicons. Interestingly, there was a difference in the sensitivity of the species-specific COI primers compared to those targeting the 16S gene. This could be explained by differences in primer efficiency, i.e. primer rating which was calculated in PrimerPremier5 (Premier Biosoft International, Paolo Alto, CA, USA). This rating takes secondary structures such as hairpins, dimers, cross dimers as well as the primer binding capacity into account which represents the primers’ efficiency to bind to complimentary DNA sequences. The average primer rating for the COI and 16S primers was 73.2 ± 13.1 and 62.9 ± 17.7 (mean ± SD), respectively, suggesting that the higher sensitivity of the COI primers is due to primer quality. For facultative endosymbiont-specific primers, although the sensitivities were higher in the 18SMP than in the 16SMP, both MP-PCR assays were sensitive enough to detect *H. defensa* in aphid mummies. In the field-collected samples, we detected one more sample each positive for *H. defensa* and *R. insecticola* using the 18SMP assay than with the 16SMP. An explanation for this could be that these were freshly *H. defensa*- and *R. insecticola*-infected samples, containing only a low concentration of bacterial DNA. The sensitivities of the aphid primers have intentionally been chosen to be much lower compared to those of all other targets to avoid a PCR in favour of amplifying aphid DNA due to the high aphid template copy number, which can prohibit the detection of small quantities of parasitoid DNA^31^. This situation is different in aphid mummy samples, where nearly the entire aphid has been consumed by the parasitoid larva so that there is relatively less aphid than parasitoid DNA present. In this case, we suggest raising the aphid primer concentration and reducing that of the parasitoid primers in the first step MP-PCRs to allow for the identification of the aphid host.

In the 18SMP assay, a single pair of 18S-based parasitoid primers was sufficient to target all parasitoid species examined in this study. For the same purpose, three pairs of 16S-based parasitoid group primers were necessary in the 16SMP. The 18S is more conserved in parasitoids, making it impossible to identify these species based on this gene. In contrast, the 16S exhibits sufficient variation to allow discrimination between most of the parasitoid species, except for the three species of *Aphidius*, as well as two species of *Aphelinus*, *Praon, Alloxysta* and *Pachyneuron*, respectively (see Supplementary Table S2) (*cf* Derocles, *et al*.^35^; Ye, *et al*.^40^). For species detection via MP-PCR, most hyperparasitoid species-specific primers target the 16S, due to the high intra-specific variation of the COI among these species^40, 56^.

Although multiplex PCR provides a high through-put and highly sensitive method with a high degree of resolution^49^, it also has its limits when it comes to the analysis of an unknown species spectrum. In this case, DNA barcoding approaches, such as Sanger and NGS may provide an integrated picture of the entire parasitoid spectrum, which is especially important at the presence of rare or unexpected species. Such DNA sequence-based approaches require a comprehensive DNA barcode database based on expertly-identified species. For the aphid-parasitoid system, this has recently been provided by Ye, *et al*.^40^, thereby allowing the identification of many parasitoid species (including those not directly targeted in the MP-PCR) via DNA sequencing. Finally, to filter out the parasitized aphids for sequence-based identification, general parasitoid primers are needed, that result in an amplicon that provides a species-specific resolution for DNA barcoding, which fully applies for the parasitoid general primer pairs developed for the 16SMP assay.

In conclusion, this two-step molecular approach provides a powerful means to identify cereal aphid-primary parasitoid-hyperparasitoid-endosymbiont interactions on a species-specific level in Central Europe. The first step MP-PCR assays allow for a quick and reliable assessment of parasitism and endosymbiont infection rates, and also provides amplicons which can be used for parasitoid identification of species not included in the second step multiplex PCR assays via a DNA sequencing approach. The first step MP-PCR assays and the respective general parasitoid primer pairs hold the potential to be adapted for other aphid-primary parasitoid-hyperparasitoid systems as the general parasitoid primers amplify also non-cereal aphid parasitoid taxa.

## Methods

### Source of aphids, parasitoids and endosymbionts

#### Aphids

Individuals of the three most important cereal aphid species in Central Europe^57^ were collected in winter wheat fields around Göttingen (Germany) in 2009, 2012 and 2013: the English grain aphid *S. avenae*, the rose-grain aphid *M. dirhodum*, and the bird cherry-oat aphid *R. padi*. Additional un-parasitised aphids belonging to *S. avenae* and *R. padi* were obtained from Katz Biotech AG (Baruth, Germany). Aphids collected in 2009 and obtained from Katz Biotech AG were identified to species level and used as voucher specimens for molecular analysis. Aphids collected in 2012 and 2013 were used to test the newly developed molecular approaches (see below).

#### Parasitoids

Adult specimens of aphid primary and hyperparasitoids species were collected at different locations in Europe (Table 4, taxonomic authorities and specimen information/providers are provided in Supplementary Tables S3 and S5). In total, 43 primary parasitoid and hyperparasitoid species were obtained. Additionally, *A. abdominalis* and *Aphidius colemani* were purchased from Sautter & Stepper GmbH (Ammerbuch, Germany) and Katz Biotech AG, respectively. All parasitoids were individually stored in 98% ethanol and morphologically identified (identification keys see Supplementary Appendix S2). For *Toxares deltiger* and *Praon necans*

**Table 4:** Number of COI, 16S, 18S sequences of the aphids, parasitoids and endosymbiont used for primer design and barcode database, including the trophic level, family and species, as well as the number of DNA sequences from specimens, collected from GenBank, and the total number of DNA sequences per gene.

Organism group	Family/Subfamily	Species	Number of COI sequences			Number of 16S sequences			Number of 18S sequences		
			from specimens	from GenBank	Total	from specimens	from GenBank	Total	from specimens	from GenBank	Total
Aphid	Aphididae	*Metopolophium dirhodum*	1	4	5				2	1	3
		*Rhopalosiphum padi*	1	6	7		2	2	3	2	5
		*Sitobion avenae*	1	6	7		2	2	2	1	3
Primary parasitoid	Aphelinidae	*Aphelinus abdominalis*	5	2	7	4		4	2		2
		*Aphelinus asychis*	1		1	1	2	3	1	1	2
		*Aphelinus chaonia*	3		3	3		3	3		3
		Aphelinus mali*	3		3	2		2	1		1
		*Aphelinus varipes*	2	4	6	1	2	3	1		1
	Aphidiinae	*Adialytus ambiguus*	1	9	10	2		2	2	1	3
		*Aphidius avenae*	5	7	12		2	2	4		4
		*Aphidius colemani*	2	9	11		3	3	2	1	3
		*Aphidius ervi*	7	8	15	2	5	7	3	1	4
		*Aphidius matricariae*	3	2	5	2	3	5	3	1	4
		Aphidius microlophii*	4	3	7		2	2	3		3
		*Aphidius rhopalosiphi*	10	19	29	3	2	5	6	1	7
		Aphidius rosae*	2	2	4		3	3	2	1	3
		Aphidius urticae*	1	3	4		2	2	1		1
		*Aphidius uzbekistanicus*	2	4	6		2	2	2		2
		*Binodoxys angelicae*		2	2		2	2	1	1	2
		Diaeretiella rapae**	10	5	15		9	9	3	1	4
		Ephedrus persicae**	3		3	2	1	3	2	1	3
		*Ephedrus plagiator*	5	4	9		3	3	4		4
		*Lipolexis gracilis*	1	2	3		3	3	2	1	3
		*Lysiphlebus fabarum*	5	25	30		9	9	3	1	4
		*Lysiphlebus testaceipes*	5	11	16	2	6	8	2	2	4
		*Monoctonus crepidis*	3	3	6		2	2	2		2
		*Praon abjectum*	2	3	5	2		2	1		1
		Praon bicolor*	3	1	4	2	2	4	3		3
		*Praon gallicum*	5	5	10		2	2	2		2
		Praon longicorne*	1		1	2		2	1		1
		*Praon necans*	2		2	3	1	4	4		4
		*Praon volucre*	4	13	17		10	10	2	1	3
		*Toxares deltiger*	1	2	3	1		1	1		1
		Trioxys auctus**	1		1	1		1	2		2
Hyperparasitoid	Encyrtidae	*Syrphophagus aphidivorus*	5	6	11				2		2
	Figitidae	*Alloxysta brachyptera*		1	1						
		*Alloxysta brevis*	2		2	1		1	2		2
		*Alloxysta fulviceps*	3	3	6	2		2	2		2
		*Alloxysta pedestris*	1	1	2	1		1	1		1
		*Alloxysta victrix*	7	2	9	4		4	4		4
		*Phaenoglyphis villosa*	5	1	6	5		5	3		3
	Megaspillidae	*Dendrocerus carpenteri*	5	3	8	4	2	6	2	1	3
		*Dendrocerus laticeps*	2		2	2		2	2		2
	Pteromalidae	*Asaphes suspensus*	6	1	7	5		5	3	1	4
		*Asaphes vulgaris*	7	9	16	4		4	3		3
		*Coruna clavata*	3	1	4	3		3	2	1	3
		*Pachyneuron aphidis*	6	7	13	3		3	4		4
		*Pachyneuron formosum*	1		1	1		1		1	1
		*Pachyneuron muscarum*	3		3	3		3	2		2
		*Pachyneuron solitarium*	1		1	2		2	3		3
Endosymbiont		*Buchnera aphidicola*					10	10			
		*Hamiltonella defensa*					7	7			
		pea aphid X-type symbiont					2	2			
		*Regiella insecticola*					7	7			
		*Serratia symbiotica*					9	9			

Non-cereal aphid parasitoids are marked with *; parasitoid species which attack cereal aphids on their winter plant host are marked with **.

, DNA extracts were directly provided by Prof. Zeljko Tomanovic (University of Belgrade, Serbia).

#### Endosymbionts

Pea aphids, *Acyrthosiphon pisum*, which were infected with different facultative endosymbionts (*H. defensa*, or *R. insecticola*, or both *H. defensa* and PAXS), mummified *Aphis fabae* individuals (parasitised by *Lysiphlebus fabarum*), which were either infected or un-infected with *H. defensa*, and a culture of *Serratia symbiotica*, another facultative aphid endosymbiont, were kindly provided by Dr. Julia Ferrari (University of York, United Kingdom), Prof. Christoph Vorburger (ETH Zürich & EAWAG, Switzerland), and Dr. Ahmed Sabri (University of Liege, Belgium), respectively. The facultative endosymbiont *S. symbiotica* was used as a non-target species, as there is limited evidence of a parasitoid defensive function associated with this species^37^.

All aphids and mummies were freeze-killed and stored individually either in 96 well plates at −80 °C (field-collected aphids) or in 98% ethanol (non-field collected aphids). In addition, a variety of parasitoid adults (Braconidae, Ceraphronidae, Diapriidae, Encyrtidae, Eucoilidae, Eulophidae, Eurytomidae, Ichneumonidae, Megaspillidae, Mymaridae, Platygastridae, Proctotrupidae, Pteromalidae, Scelionidae and Trichogrammatidae) that are not associated with aphids were collected by sweep-netting in and around the experimental fields in 2012 or provided by Dr. Daniela Sint (University of Innsbruck, Austria); these were used as test species to validate the specificity of the primers developed for aphid parasitoids.

### DNA extraction

Cereal aphids collected in 2009 or obtained from Katz Biotech AG, *A. pisum* infected with different facultative endosymbionts (*H. defensa*, or *R. insecticola*, or both *H. defensa* and PAXS), as well as the culture of *S. symbiotica* were DNA-extracted using the DNeasy Blood & Tissue Kit (Qiagen, Hilden, Germany) or the BioSprint 96 DNA Blood Kit (Qiagen) following the manufacturer’s instructions. All parasitoid adult were non-destructively lysed in 180 µl ATL buffer (Qiagen) and 20 µl proteinase K (20 mgml^−1^, AppliChem, Darmstadt, Germany) for 2 h. A non-destructive method was used to keep the individuals intact for additional morphological identification, if needed. The lysates were DNA-extracted using the two extraction methods described above.

The mummified *A*. *pisum* individuals and the aphids from both the parasitism experiment (Supplementary Appendix S4) and the field collections (collected in 2012 and 2013), were DNA-extracted as follows: per aphid, 25 DNA-free glass balls (Ø 1 mm), 20 µl phosphate buffered saline (PBS PH 7.2; Sigma-Aldrich, St. Louis, MO, USA), and 5 µl Proteinase K (20 mgml^−1^, AppliChem) were added. Samples were ground twice using a ball mill (MM301, Retsch, Haan, Germany) for 40 s at 30 Hz. After a quick spin at 13,000 rpm, 150 µl of 10% Chelex (Bio-Rad, Hercules, CA, USA) solution was added followed by an overnight incubation at 56 °C. Finally, the samples were incubated at 95 °C for 15 min and then stored at −28 °C until PCR. Within every batch of 96 samples, at least two extraction negative controls were included to check for potential DNA cross-contamination. These negative extraction controls were tested using the first step MP-PCR assays.

### Sequencing and primer design

#### DNA Sequencing

In order to develop group-specific primers for various parasitoid genera associated with aphids (see Table 4), the 18S ribosomal RNA gene (18S) or 16S ribosomal RNA gene (16S) was sequenced. In addition, partial 16S and cytochrome *c* oxidase subunit I gene (COI) sequences were generated in order to develop species-specific aphid and parasitoid primers for selected species (see Table 1). Details for general primers and PCR conditions are described in Supplementary Appendix S3. DNA was sequenced bi-directionally at Eurofins MWG Operon (Ebersberg, Germany). The DNA sequences were checked, assembled and aligned using BioEdit sequence alignment editor 7.0.0^58^. Additionally, COI, 16S and 18S sequences of cereal aphids and parasitoids were obtained from GenBank and included in the database used for primer design (Table 4, GenBank accession numbers see Supplementary Table S4).

#### Group-Specific Parasitoid Primers

Group-specific primers were developed with the intent to amplify all parasitoid genera associated with aphids. One pair of a parasitoid group-specific primer was designed based on partial 18S sequences. Another three pairs of primary parasitoid and hyperparasitoid group-specific primers were generated based on partial 16S sequences: The first primer pair covers all primary parasitoids within the Aphidiinae (Hymenoptera: Braconidae), the second one covers members of the genus *Aphelinus* and all hyperparasitoid species except for the genus *Dendrocerus*. These two 16S parasitoid group-specific primer pairs share the same reverse primer, an optimized version of the Aphidiinae primer 16S-Rspe^35^. The third parasitoid group-specific primer pair covers *D. carpenteri* and *Dendrocerus laticeps*.

#### Species-Specific Primer Pairs

Additionally, a genus-specific primer pair for *Aphidius* spp. (except for *A. avenae*), and five species-specific primer pairs for *A. abdominalis*, *A. victrix*, *A. suspensus*, *A. vulgaris* and *C. clavata* were generated based on 16S sequences. Two specific primer pairs for *R. padi*, as well as another 10 primer pairs including *S. avenae*, *M. dirhodum*, *A. ervi*, *A*. *avenae*, *A. uzbekistanicus*, *E. plagiator*, *P. gallicum*, *P. volucre*, *D*. *carpenteri* and *P. villosa* were based on partial COI sequences. Furthermore, species-specific primers for aphid facultative endosymbionts were designed based on 16S sequences (Table 4).

All primers were generated using PrimerPremier5 (Premier Biosoft International) following the guidelines for allele-specific primer design^59^.

### Multiplex PCR assays

A two-step MP-PCR approach was established (Fig. 1): in the first step, DNA of all primary and hyperparasitoid species was targeted using general primers to detect parasitised aphid individuals. Additionally, this assay includes species-specific primers for *S*. *avenae*, *R*. *padi*, *M. dirhodum*, as well as for the facultative endosymbionts *H*. *defensa*, *R*. *insecticola*, and PAXS. In the second step, most common primary parasitoid and hyperparasitoid species in Central Europe are identified.

We developed two versions of the first step MP-PCR: the 18S MP-PCR assay (18SMP) includes one pair of 18S parasitoid group-specific primers as well as the species-specific primer pairs for the three aphid species and the facultative endosymbiont species. Whereas the 16S MP-PCR assay (16SMP) contains three pairs of mitochondrial 16S parasitoid group-specific primers (one for the Aphidiinae, one for *Aphelinus* and most hyperparasitoids, and another one for two *Dendrocerus* species), which allows the identification to be further refined, and the appropriate second step to be selected (see below). Except for the *R*. *padi* primer pair, aphid and facultative endosymbiont species-specific primers in this MP-PCR are the same as in the 18SMP. A new *R. padi* primer pair was used in the 16SMP to avoid generating side bands as observed for the *R. padi* primer pair employed in the 18SMP.

The second step in the MP-PCRs consists of two assays: the primary parasitoid MP-PCR assay (PriMP) includes six pairs of primers targeting *A*. *ervi*, *A*. *avenae*, *A*. *uzbekistanicus*, *E*. *plagiator*, *P*. *gallicum* and *P*. *volucre*, as well as an *Aphidius* group-specific primer pair. The hyperparasitoid MP-PCR assay (HypMP) includes a pair of *A*. *abdominalis* primers, and six pairs of hyperparasitoid primers, targeting *A*. *victrix*, *A*. *suspensus*, *A*. *vulgaris*, *C. clavata*, *D. carpenteri* and *P. villosa*. The second step MP-PCR assays were applied to samples that tested positive for parasitoid DNA in the first step assays in order to further identify the parasitoids and/or hyperparasitoids detected in the first step.

Each 10 µl reaction of the first step MP-PCR assays (18SMP and 16SMP) contained 1.5 µl of extracted DNA, 5 µl of 2 × Multiplex PCR Master Mix (Qiagen), 5 µg BSA, each primer at its specific concentration (Table 1) and PCR-water to adjust the volume. The same reaction mix was used for the two second step MP-PCRs (PriMP and HypMP), but with the addition of 20 µM Tetramethylammonium chloride (TMAC; final concentration in the 10 µl reaction) to enhance the specificity of the MP-PCR assays. All MP-PCRs were carried out in a Master Cycler Gradient (Eppendorf). The thermocycling conditions were 95 °C for 15 min, following by 35 cycles of 94 °C for 30 s, 56 °C (18SMP) or 62.5 °C (16SMP) or 62 °C (PriMP) or 60.5 °C (HypMP) for 90 s, and 72 °C for 60 s, and final extension of 72 °C for 10 min.

Within each batch of samples, target DNA and molecular grade water were included as positive and negative PCR controls, respectively. All PCR products were separated and visualized using the automatic capillary electrophoresis system QIAxcel (QIAxcel DNA Screening Kit; Qiagen) and the BioCalculator Fast Analysis Software version 3.0 (method AL320). PCR products with the expected fragment lengths at a signal ≥0.1 relative fluorescent units (RFU) were counted as positives.

### Identification of parasitoids not covered by the second step MP-PCR assays via DNA sequencing

The second-step MP-PCR assays allows the identification of 13 species and one genus of parasitoids, which were among the most common species associated with aphids in Central Europe. Aphid parasitoids which are less commonly encountered were identified by comparison of their 16S DNA sequence with the parasitoid 16S sequences available in GenBank and our newly generated sequence database (Table 4; GenBank accession numbers of the sequences see Supplementary Tables S4 and S5). Sequences were generated using the three 16S-based parasitoid group-specific primer pairs of the 16SMP assay in singleplex PCRs. Each 10 µl singleplex PCR contained 1.5 µl of extracted DNA, 5 µl of 2× Multiplex PCR Master Mix (Qiagen), 5 µ BSA, 1 µM of each primer and PCR-water to adjust the volume. Thermocycling and visualization of PCR products was conducted as described for the 16SMP above. Purification and sequencing was as described in Supplementary Appendix S3.

### Assay sensitivity and specificity as well as testing of field-collected samples

#### Assay sensitivity and primer balancing

For determining the sensitivity of the group-specific parasitoid primer pairs in the first step MP-PCR assays, parasitised aphids collected at 1 h, 1 d, 2 d, 3 d, 5 d, and 7 d post-parasitism were used. Afterwards, the sensitivities of the three parasitoid primer pairs employed in the 16SMP were balanced using standardised DNA templates (for the 18SMP this was not done as only one parasitoid primer pair is included). For determining the sensitivity of parasitoid detection in the second step MP-PCR assays, the minimum number of template DNA per target detectable was determined. To check if endosymbiont DNA can be detected in mummified aphids, 10 mummies each of *H. defensa*-infected and uninfected *A. pisum* which contained *L. fabarum* as parasitoid (“full mummy”) were tested. Additionally, to assess if endosymbiont DNA is still detectable when the parasitoid has emerged, five “empty mummy” samples were used. For each of these samples we extracted the DNA from the empty mummy plus the parasitoid adult. These DNA extracts should thus contain a low quantity of endosymbiont DNA but a high quantity of parasitoid DNA. The facultative endosymbiont and aphid-specific primers were balanced using standardised DNA templates. Details of this part see Supplementary Appendix S4.

#### Assay Specificity

The specificity of the MP-PCR assays was tested with the targeted aphid, parasitoid and endosymbiont species. Additionally, the first step MP-PCR assays were tested for primer generality with the non-cereal aphid parasitoid species. In the second step MP-PCR assays, the three cereal aphid species, 27 less common parasitoid species, as well as four facultative endosymbiont species were used for non-target testing. Details of this part see Supplementary Appendix S5.

#### Field sample tests

The newly-developed multiplex PCR assays were applied to DNA extracts of field-collected aphids (n = 42) and aphid mummies (n = 48) collected by hand in 2012 and 2013 (see above), to validate their utility for the detection and identification of parasitoid, hyperparasitoid, and endosymbiont communities in nature.

### Data Accessibility

All the sequences generated in this study were submitted to GenBank. GenBank accession numbers: KY873325 to KY873374, KY887805 to KY887993, and KY912635 to KY912707.

## Electronic supplementary material

Supplementary information 1

Supplementary information 2
