# Supplementary material for: An effective molecular approach for assessing cereal aphid-parasitoid-endosymbiont networks
Source: Sci Rep. 2017 Jun 9;7:3138. doi: 10.1038/s41598-017-02226-w (PMC5466676; doi:10.1038/s41598-017-02226-w)
Supplement: Supplementary file 1 — Supplementary information 1 [file 41598_2017_2226_MOESM1_ESM.pdf]

An effective molecular approach for assessing cereal aphid-parasitoid-endosymbiont networks

Zhengpei Ye[1*], Ines Vollhardt[2], Susanne Girtler[1], Corinna Wallinger[1], Zeljko Tomanovic[3] & Michael Traugott[1]

[1] *Mountain Agriculture Research Unit, Institute of Ecology, University of Innsbruck, Austria*

[2] *Agroecology, Department of Crop Sciences, Georg-August-University Göttingen, Germany*

[3] *Institute of Zoology, Faculty of Biology, University of Belgrade, Serbia*

***Corresponding author:**

Zhengpei Ye

Mountain Agriculture Research Unit, Institute of Ecology, University of Innsbruck, Technikerstrasse 25, 6020 Innsbruck, Austria

Phone: +43 (0)512 507-51676, Fax: +43 (0)512 507-51799; E-Mail: Zhengpei.Ye@uibk.ac.at

Appendix S1: unexpected PCR products in 16SMP and their interpretation

The *Dendrocerus* forward primer and the shared reserve primer ParG-A462 produce an additional 149/150 bp amplicon when DNA of *Dendrocerus* was present. This amplicon is similar in size to the *Aphelinus*-hyperparasitoid PCR product (155 ± 2 bp). This side band, however, does not corrupt the detection of either *Aphelinus* parasitoids or hyperparasitoids, because the samples, which score *Aphelinus*-hyperparasitoid- or *Dendrocerus*-positive, are subjected to the second step HypMP assay anyway. Secondly, all *Dendrocerus* spp. parasitoid samples provide a weak and unexpected side band which is of similar size as the one for *S. avenae* (259 bp). However, this side band, which is only amplified at the presence of a large amount of *Dendrocerus* DNA, such as in a DNA extract of an adult parasitoid, does not cause problems for correct aphid species identification: if this *Dendrocerus* side band occurs together with the bands specific for one of the two other aphid species (*R. padi* and *M. dirhodum*, respectively), the individual can be identified as the respective aphid species. In case of *S .avenae*, there may just be a thicker band comprising both the *S. avenae* specific band and the sideband.

Appendix S2: Morphological identification keys

The parasitoids were morphologically identified by specialists using multiple taxonomic keys[1-15].

Appendix S3: General primers and PCR conditions used to generate COI, 16S and 18S sequences

The 18S sequences of aphids and parasitoids were amplified and sequenced using the general primers 18SL0001 and 18SR1100[16]. The 16S sequences of aphids and parasitoids were amplified and sequenced using general primers '5'-CGCCGTTTTATCAAAAACATGT-3'', a modification of the LR-N-13398 primer[17] by Kambhampati, et al. [18] and LR-J-13017[19] or LR-N13398 version '5'-CACCTGTTTATCAAAAACAT-3''[17] and LR-J12888 version '5'-TCGATTTGAACTCARATCATGTA-3''[20]. The COI fragments of aphids and parasitoids were amplified and sequenced using the general invertebrate primers LCO-1490 and HCO-2198[21]. For PCR, 10 µl reactions contained 1.5 µl template DNA, 5 µl of 2 × Multiplex PCR Master Mix (Qiagen,

Hilden, Germany), 1 µM of each of the respective primers and PCR-water to adjust the volume. The PCRs were carried out in a Master Cycler Gradient (Eppendorf, Hamburg, Germany) using the following thermocycling conditions: 95 °C for 15 min, 35 cycles of 94 °C for 30 s, 50 °C for 90 s, 72 °C for 60 s, and a final extension of 72 °C for 10 min. PCR products were visualized on 1.5 % agarose gels, purified with ExoSap-IT (Amersham Biosciences, Glattbrugg, Switzerland) following the manufacturer's instructions, and sent to Eurofins MWG Operon (Ebersberg, Germany) for bi-directional sequencing.

Appendix S4: Multiplex PCR assay sensitivity testing and primer balancing

Parasitism experiment: Live *S. avenae* (host) and *L. testaceipes* (parasitoid) were obtained from Katz Biotech AG. Aphids were kept on young wheat plants inside fine mesh cages in a climate chamber (~25 ± 1℃, 12:12h L:D), separated from a colony of *L. testaceipes* maintained on *S. avenae* at similar conditions. Unparasitised *S. avenae* nymphs were individually placed in 1.5 ml centrifuge tubes with one female *L. testaceipes*. After oviposition was observed, the aphids were transferred onto a fresh young wheat plant inside a mesh cage in the climate chamber. To test how early parasitoid DNA can be detected within the aphids, batches of at least 15 parasitised aphids each were collected from the plants at 1 h, 1 d, 2 d, 3 d, 5 d, and 7d post-parasitism, and individually frozen at -80°C. Additionally, 15 un-parasitised aphids and 15 parasitoids were frozen to serve as negative and positive controls in the molecular testing, respectively.

Generating standardised DNA templates: For determining the sensitivity of the MP-PCR assays and balancing amplification efficiencies between primer pairs, standardised DNA templates of each targeted taxon were generated following the approach described in Sint, et al. [22]. 16S templates of *Aphidius ervi*, *Lysiphlebus testaceipes*, *Aphidius rhopalosiphi*, *Alloxysta victrix*, *Asaphes suspensus*, *Asaphes vulgaris* and *Coruna clavata* were amplified using the general primers LR-N13889 '5'-CACCTGTTTATCAAAAACAT-3'' [17] and LR-J12888 '5'-TCGATTTGAACTCARATCATGTA-3'' [20]. For the facultative endosymbionts *Hamiltonella defensa* and *Regiella insecticola*, 16S templates were amplified using the modified general forward and reverse primers for facultative aphid endosymbionts, 10F '5'-GAGTTTGATCATGGCTCAGATTG-3'' [23] and 35R[24], respectively. 16S

templates of the facultative endosymbiont pea aphid X-type symbiont (PAXS) were amplified by the newly designed forward primer PAXS-S494 '5'-GGCACGTAGGCGGTTTC-3'' and the already published reverse primer 35R[24]. COI templates of cereal aphids (*Sitobion avenae*, *Rhopalosiphum padi* and *Metopolophium dirhodum*) and parasitoids (*A. ervi*, *Aphidius avenae*, *Aphidius uzbekistanicus*, *Ephedrus plagiator*, *Praon gallicum*, *Praon volucre*, *Dendrocerus carpenteri* and *Phaenoglyphis villosa*) were amplified using the general invertebrate primers LCO-1490 and HCO-2198[21]. For PCR, 20 µl reactions contained 3 µl template DNA, 10 µl of 2 × Multiplex PCR Master Mix (Qiagen, Hilden, Germany), 2 µM of each of the respective primers and PCR-water to adjust the volume. The PCRs were carried out in a Master Cycler Gradient (Eppendorf, Hamburg, Germany) using the following thermocycling conditions: 95 °C for 15 min, 35 cycles of 94 °C for 30 s, 50 °C for 90 s, 72 °C for 60 s, and a final extension of 72 °C for 10 min. PCR template concentrations were standardised and serially diluted to 10,000, 5,000, 1,000, 500, 100, 50, 10 and 5 double-stranded DNA templates/µl.

To test the sensitivity of the first step MP-PCRs, the aphids from the parasitism experiment were checked for parasitoid DNA to confirm parasitisation success, using a PCR protocol allowing to detect a freshly inserted parasitoid egg already[25]. All aphid samples which were tested positive in this assay were deemed to contain a parasitoid egg or larva.

The two first step MP-PCR assays were tested with the *L. testaceipes*-positive samples, using 10 replicates per time point post parasitism. Afterwards, the sensitivities of the three parasitoid primer pairs employed in the 16SMP were balanced using standardised DNA templates (for the 18SMP this was not done as only one parasitoid primer pair is included). First, the number of *L. testaceipes* DNA templates that allow the detection of eggs of this parasitoid species by the Aphidiinae primer pair was determined; thereafter, the sensitivities of *Aphelinus*-hyperparasitoid and *Dendrocerus* spp. group-specific primer pairs were adjusted to the same sensitivity as the Aphidiinae primer pair by adjusting the primers' concentrations using DNA templates of *A. suspensus* and *D. carpenteri*, respectively. As *L. testaceipes* is not a common species in the experimental region, the 16SMP was also tested with known numbers of DNA templates of *A. ervi* to assure that the Aphidiinae primer pair has similar sensitivity for different Aphidiinae species. For the sensitivity-testing of species-specific parasitoid

identification in the second step MP-PCR assays, the minimum number of template DNA per target species detectable was determined. For the *Aphidius* spp. genus-specific primer pair 16S templates of *A. rhopalosiphi* were used.

For the facultative endosymbiont and aphid specific primers included in the two first step MP-PCR assays, the process of sensitivity testing and primer balancing was conducted using the same strategy as described above. To check if facultative endosymbiont DNA is detectable in mummified aphids, 10 aphid mummies each from both a *H. defensa*-infected and a *H. defensa*-free population of *A. pisum* were tested with the two first-step MP-PCR assays. To test if facultative endosymbiont DNA is detectable even after the parasitoid adult emerges, five *H. defensa*-infected mummified *A. pisum* samples were kept until the parasitoid adult emerged. Thereafter, the empty mummy and the respective adult parasitoid were DNA-extracted as one sample. These five samples were tested with the two first step MP-PCR assays. After the determination of the minimum number of DNA template of *H. defensa* detectable by these assays, the concentrations of *R. insecticola* and PAXS primer pairs were adjusted accordingly to obtain similar detection sensitivity.

To ensure high assay sensitivity for the detection of parasitoids and facultative endosymbionts in the first step MP-PCRs, the concentration of the aphid primers was reduced to avoid that these primers dominate the PCR when high concentrations of aphid DNA is present in the sample, i.e. when a freshly parasitised/endosymbiont infected aphid is examined.

Appendix S5: Multiplex PCR assay specificity testing

The specificity of the MP-PCR assays was tested with the targeted aphid, parasitoid and endosymbiont species. All aphids were checked for the presence of endosymbiont DNA according to Ferrari, et al. [26], with *Serratia symbiotica* as a non-target. Additionally, the first step MP-PCR assays were tested for primer generality with at least two individuals of each family of the non-cereal aphid parasitoids, except with only one specimen for Trichogrammatidae. In the second step MP-PCR assays, the three cereal aphids, 28 less common parasitoids, as well as four facultative endosymbionts were used for non-target testing.

Generally, at least three individuals per species were used for specificity testing, for the parasitoids *Aphelinus asychis*, *Aphelinus varipes*, *Aphidius rosae*, *Alloxysta brachyptera* and *Dendrocerus laticeps*. For *Toxares deltiger*, *Alloxysta pedestris* (n = 2), and *Pachyneuron formosum* (n = 1). In addition, two PCR products per target taxon of field-collected samples were sequenced to confirm the species identity. Only one PCR product each was obtained for *Asaphes suspensus* and *Coruna clavata*, respectively, and no pea aphid X-type symbiont (PAXS) at all.

Table S1: Number of amplicons generated in non-cereal aphid parasitoids using the first MP-PCR assays (18SMP and 16SMP).

Organism group	Family	Species	Number of individuals	Number of positive amplicons		
				**General parasitoid (18SMP)**	***Aphelinus*-Hyperparasitoid (16SMP)**	**Aphidiinae (16SMP)**
Parasitoids	Braconidae	*Apanteles subandinus*	1	1		
		Chorebus sp.	1	1		
		Cotesia glomerata	1	1	1	
		Cotesia rubecula	1	1	1	
		Dolichogenidea gelechiidivoris	1			1
		Microplitis mediator	1	1		1
		Opius dissitus	1			
		Orgilus lepidus	1	1	1	
		Phaedrotoma scabriventris	1	1		
		unidentified	1			
	Ceraphronidae	*Aphanogmus* sp.	1	1		
		Ceraphronus sp.	1			
	Diapriidae	unidentified	2	2		
	Encyrtidae	*Copidosoma koehleri*	1	1	1	
		unidentified	1	1	1	
	Eucoilidae	unidentified	2	2		
	Eulophidae	*Chrysocharis caribea*	1	1		
		Chrysocharis flacilla	1	1	1	
		Diglyphus sp.	1	1	1	
		Diglyphus websteri	1	1	1	
		Pnigalio sp.	1	1	1	
	Eurytomidae	unidentified	2	2	2	
	Ichneumonidae	*Aritranis director*	1	1		
		Diadegma semiclausum	1	1	1	

		unidentified	2	1	
	Megaspillidae	*Archiphauurus* sp.	1	1	
		Conostigmus sp.	1	1	
	Mymaridae	unidentified	2	2	
	Platygastridae	*Euxestonotus* sp.	1	1	
		Leptacis sp.	1		
	Proctotrupidae	Unidentified	2	1	2
	Pteromalidae	*Halticoptera arduine*	1	1	1
		unidentified	2	2	2
	Scelionidae	*Trissolcus* spp.	3	3	
	Trichogrammatidae	*Trichogramma* sp.	1	1	1
Other Hymenoptera	Apidae	*Apis mellifera*	1	1	
		Bombus pascuorum	1	1	1
	Symphyta	unidentified	1	1	
	Vespidae	*Polistes dominula*	1	1	

Table S2: Five parasitoid groups have no between species distances within group on partial 16S sequences, which are generated using the 16S general parasitoid primers in 16SMP. Non-cereal aphid parasitoids are marked with *; parasitoid species which attack cereal aphids on their winter plant host are marked with **.

**Trophic level**	**Family/Subfamily**	**Species-group**
Primary Parasitoid	Aphelinidae	*Aphelinus chaonia* & *Aphelinus varipes*
	Aphidiinae	*Aphidius ervi, Aphidius microlophii*, Aphidius rhopalosiphi, Aphidius urticae** & *Aphidius uzbekistanicus*
		Aphidius matricariae & *A. urticae**
		*Praon abjectum, Praon bicolor** & *Praon volucre*
Hyperparasiotid	Pteromalidae	*Pachyneuron muscarum* & *Pachyneuron solitarium*

Table S3: Taxonomic authorities of the organisms used in this study.

Organism group	Family	Species/Genus	Taxonomic authority
Aphid	Aphididae	*Acyrthosiphon pisum*	Harris 1776
	Aphididae	*Aphis fabae*	Scopoli 1763
	Aphididae	*Metopolophium dirhodum*	Walker 1849
	Aphididae	*Rhopalosiphum padi*	L. 1758
	Aphididae	*Sitobion avenae*	Fabricius 1775
Aphid primary parasitoid	Aphelinidae	*Aphelinus abdominalis*	Dalman 1820
	Aphelinidae	*Aphelinus asychis*	Walker 1839
	Aphelinidae	*Aphelinus chaonia*	Walker 1839
	Aphelinidae	*Aphelinus mali*	Haldeman 1851
	Aphelinidae	*Aphelinus varipes*	Förster 1841
	Braconidae	*Adialytus ambiguus*	Haliday 1834
	Braconidae	*Aphidius avenae*	Haliday 1834
	Braconidae	*Aphidius colemani*	Viereck 1912
	Braconidae	*Aphidius ervi*	Haliday 1834
	Braconidae	*Aphidius matricariae*	Haliday 1834
	Braconidae	*Aphidius microlophii*	Pennacchio & Tremblay 1987
	Braconidae	*Aphidius rhopalosiphi*	Stefani-Perez 1902
	Braconidae	*Aphidius rosae*	Haliday 1834
	Braconidae	*Aphidius urticae*	Haliday 1834
	Braconidae	*Aphidius uzbekistanicus*	Luzhetzki 1960
	Braconidae	*Binodoxys angelicae*	Haliday 1833
	Braconidae	*Diaeretiella rapae*	M'Intosh 1855
	Braconidae	*Ephedrus persicae*	Froggatt 1904
	Braconidae	*Ephedrus plagiator*	Nees 1811
	Braconidae	*Lipolexis gracilis*	Förster 1862
	Braconidae	*Lysiphlebus fabarum*	Marshall 1896
	Braconidae	*Lysiphlebus testaceipes*	Cresson 1880
	Braconidae	*Monoctonus crepidis*	Haliday 1834
	Braconidae	*Praon abjectum*	Haliday 1833
	Braconidae	*Praon bicolor*	Mackauer 1959
	Braconidae	*Praon gallicum*	Starý 1971
	Braconidae	*Praon longicorne*	Starý 1971
	Braconidae	*Praon necans*	Mackauer 1959
	Braconidae	*Praon volucre*	Haliday 1833
	Braconidae	*Toxares deltiger*	Haliday 1833
	Braconidae	*Trioxys auctus*	Haliday 1833
Hyperparasiotid	Encyrtidae	*Syrphophagus aphidivorus*	Mayr 1876
	Figitidae	*Alloxysta brachyptera*	Hartig 1840
	Figitidae	*Alloxysta brevis*	Thomson 1862
	Figitidae	*Alloxysta fulviceps*	Curtis 1838
	Figitidae	*Alloxysta pedestris*	Curtis 1838
	Figitidae	*Alloxysta victrix*	Westwood 1833
	Figitidae	*Phaenoglyphis villosa*	Hartig 1841
	Megaspillidae	*Dendrocerus carpenteri*	Curtis 1829

	Megaspillidae	*Dendrocerus laticeps*	Hedicke 1929
	Pteromalidae	*Asaphes suspensus*	Nees 1834
	Pteromalidae	*Asaphes vulgaris*	Walker 1834
	Pteromalidae	*Coruna clavata*	Walker 1833
	Pteromalidae	*Pachyneuron aphidis*	Bouché 1834
	Pteromalidae	*Pachyneuron formosum*	Walker 1833
	Pteromalidae	*Pachyneuron muscarum*	L. 1758
	Pteromalidae	*Pachyneuron solitarium*	Hartig 1838
non-aphid parasitoid	Braconidae	*Apanteles subandinus*	Blanchard 1947
	Braconidae	*Chorebus* sp	Haliday 1833
	Braconidae	*Cotesia glomerata*	L. 1758
	Braconidae	*Cotesia rubecula*	Marshall 1885
	Braconidae	*Dolichogenidea gelechiidivoris*	Marsh 1979
	Braconidae	*Microplitis mediator*	Haliday 1834
	Braconidae	*Opius dissitus*	Muesebeck 1963
	Braconidae	*Orgilus lepidus*	Muesebeck 1967
	Braconidae	*Phaedrotoma scabriventris*	
	Ceraphronidae	*Aphanogmus* sp	Thomson 1858
	Ceraphronidae	*Ceraphronus* sp	Jurine 1807
	Encyrtidae	*Copidosoma koehleri*	Blanchard 1940
	Eulophidae	*Chrysocharis caribea*	Boucek 1977
	Eulophidae	*Chrysocharis flacilla*	Walker 1842
	Eulophidae	*Diglyphus* sp	Walker 1844
	Eulophidae	*Diglyphus websteri*	Crawford 1912
	Eulophidae	*Pnigalio* sp	Schrank 1802
	Ichneumonidae	*Aritranis director*	Thunberg 1824
	Ichneumonidae	*Diadegma semiclausum*	Hellén 1949
	Megaspillidae	*Archiphanurus* sp	
	Megaspillidae	*Conostigmus* sp	Dahlbom 1858
	Platygastridae	*Euxestonotus* sp	Fouts 1925
	Platygastridae	*Leptacis* sp	Foerster 1856
	Pteromalidae	*Halticoptera arduine*	Walker 1843
	Scelionidae	*Trissolcus* sp	Ashmead 1893
	Trichogrammatidae	*Trichogramma* sp	Westwood 1833
other Hymenoptera	Apidae	*Apis mellifera*	L. 1758
	Apidae	*Bombus pascuorum*	Scopoli 1763
	Vespidae	*Polistes dominulus*	Christ 1791

Table S4: Aphid parasitoid and endosymbiont DNA sequences retrived from GenBank for this study. Non-cereal aphid parasitoids are marked with *; parasitoid species which attack cereal aphids on their winter plant host are marked with **.

Gene	Organism group	Family/Subfamily	Species	GenBank accession number
COI	Aphid	Aphididae	*Metopolophium dirhodum*	DQ499039, DQ499040,FN868599, KT204426
			Rhopalosiphum padi	DQ499056, EU701894, FJ009050, GU457795
			Rhopalosiphum padi	HQ979401, KT204427
			Sitobion avenae	EU701907, FN868603, GU138697, GU978931, JF806536, KT204428
	Primary Parasitoid	Aphelinidae	*Aphelinus abdominalis*	FM210123, JX507444
			Aphelinus varipes	HQ599571, JX507449, KJ086033, KJ088823
		Aphidiinae	*Adialytus ambiguus*	KJ719605, KJ719606, KJ719607, KJ719608, KJ719609, KJ719610, KJ719611, KJ719612, KJ719613
			Aphidius avenae	EU819392, EU819393, JN164785, JN620545, JN620547, JQ723406, JQ723408
			Aphidius colemani	FM210125, FM210126, JN620548, JN620549, KJ615362, KJ615370-KJ615373
			Aphidius ervi	FM210130, FM210131, FM210132, FM210134, JX507435, KC211025, KC211026, KT706472
			Aphidius matricariae	JN620562
			*Aphidius microlophii**	JN620566, JN620568, JX507434,
			Aphidius rhopalosiphi	EU819401, EU819402, EU819403-EU819406, JN164753, JN164754, JN164763, JN164777, JN164778, JN620570-JN620572, JX507437, KF597710, KJ088590, KJ615376
			*Aphidius rosae**	JN620580, JN620582
			*Aphidius urticae**	JN620590, JX507433, JX507436
			Aphidius uzbekistanicus	JN164735, JN164746, JN620594, KF597706
			Binodoxys angelicae	JF730315, JN620603
			*Diaeretiella rapae***	JF730316
			*Diaeretiella rapae***	JN620613, JN620615, KF802814
			Ephedrus plagiator	JN620623, JN620625, JN620627, JX507443
			Lipolexis gracilis	JN620635, JN620636
			Lysiphlebus fabarum	JF730314, JN620645, JN620647, JN620652, JQ723415, JX507442, KC237766-KC237768, KF597685, KF597686, KF597688-KF597690, KF597681, KF597692, KM408522, KP663444, KP663448-KP663450, KP663455-KP663457, KP663459
			Lysiphlebus testaceipes	FM210176, HQ599569, JN620653, JN620655, JX470530, JX470531, JX470533, KC237764, KC237765, KJ087120, KJ090001
			*Monoctonus caricis***	JN620657, JN620658
			Monoctonus cerasi	JX507448
			*Monoctonus crepidis**	JN620660, JN620661, JN620662
			Praon abjectum	KC128669- KC128671
			*Praon bicolor**	JN620672
			Praon gallicum	EU574906, EU819398-EU819400, JN620679
			Praon volucre	EU819394-EU819397, JN620687-JN620689, JN620681, KJ698487, KJ698496, KJ698504,

				KJ698507
			Toxares deltiger	EU819391, KP663464
	Hyperparasiotid	Encyrtidae	*Syrphophagus aphidivorus*	KF597765, KF597768, KF597770
		Figitidae	*Alloxysta brachyptera*	JX507466
			Alloxysta circumscripta	JX507461
			Alloxysta fulviceps	JX507464, JX507469, JX507470
			Alloxysta macrophadna	JX507467
			Alloxysta pedestris	JX507472
			Alloxysta victrix	EU819388, JX507475
			Phaenoglyphis villosa	JX507458
		Megaspillidae	*Dendrocerus carpenteri*	EU819389, JF906505, JX507452
		Pteromalidae	*Asaphes suspensus*	JX507454
			Asaphes vulgaris	EU819407, EU819408, JX507453, KF802812, KF802813, KM556888, KM557440, KM561413, KM565300
			Coruna clavata	JX507456
			Pachyneuron aphidis	JF906503, JX507457, KF597737, KF597738, KF597739, KF597740, KF597741
16S	Aphid	Aphididae	*Rhopalosiphum padi*	AY745781, U36743
			Sitobion avenae	AY745779, HM117805
	Primary Parasitoid	Aphelinidae	*Aphelinus asychis*	AF289137, AF289138
			Aphelinus varipes	AF289135, AF289136
			Aphidius avenae	JQ240491, KP983098
			Aphidius colemani	AF289145, JQ240494, KP983101
			Aphidius ervi	AF174310, AF176067, AF289147, GU237126, JQ240499
			Aphidius matricariae	AF289148, GU237127, JQ240509
			*Aphidius microlophii**	JQ240513, KP983113
			Aphidius rhopalosiphi	JQ240517, KP982944
			*Aphidius rosae**	AF003478, JQ240529, KP983125
			*Aphidius urticae**	JQ240539, KP982956
			Aphidius uzbekistanicus	JQ240541, KP983140
			Binodoxys angelicae	AF174334, JQ240549
			*Diaeretiella rapae***	AF174315, AF289143, AY194244, AY194245, AY194248, AY194251, AY194252, JQ240559, KP982970
			*Ephedrus persicae***	AF174348
			Ephedrus plagiator	AF176068, JQ240571, KP982994
			Lipolexis gracilis	AF174338, AF176063, JQ240581
			Lysiphlebus fabarum	AF174321, AJ005426, AY207558, JQ240592, JQ240594, JQ240596, KJ848488, KP983024, KP983043
			Lysiphlebus testaceipes	AF174323, AF289142, AY207560, AY498557, AY745773, AY745774
			*Monoctonus crepidis**	AF174339, JQ240604
			*Praon bicolor**	JQ240614, KP983155
			Praon gallicum	EU574898, JQ240620
			Praon necans	AF174353
			Praon volucre	AF174352, JQ240624, JQ240626, JQ240630, KJ848490, KP983046, KP983063, KP983070, KP983074, KP983076

	Endosymbiont		*Buchnera aphidicola*	AY518294, AY849937, FJ357459-FJ357466
			Hamiltonella defensa	AF293616, AY136141, AY296733, FJ357491, FJ357493, FJ655537, FJ655538
			pea aphid X-type symbiont	FJ821502, FJ821502
			Regiella insecticola	AY296734, AY462102, AY907547, DQ010008, FJ357495, FJ357497, FJ357498
			Serratia symbiotica	AB522706, FJ655518, FJ655519, FJ655521, FJ655523-FJ655525, FJ655530, FJ655531
18S	Aphid	Aphididae	*Metopolophium dirhodum*	KT204362
			Rhopalosiphum padi	KT204363, U27825
			Sitobion avenae	KT204364
	Primary Parasitoid	Aphelinidae	*Aphelinus asychis*	JN623060
		Aphidiinae	*Adialytus ambiguus*	AJ009317
			Aphidius colemani	AJ009318
			Aphidius ervi	AJ009321
			Aphidius matricariae	AJ009324
			Aphidius rhopalosiphi	KT204373
			*Aphidius rosae**	AJ009325
			Binodoxys angelicae	AJ009349
			*Diaeretiella rapae***	AJ009323
			*Ephedrus persicae***	AJ009329
			Lipolexis gracilis	AJ009334
			Lysiphlebus fabarum	AJ009332
			Lysiphlebus testaceipes	AJ009335, AY216698
			Praon volucre	AJ009347
	Hyperparasiotid	Megaspillidae	*Dendrocerus carpenteri*	AY918978
		Pteromalidae	*Asaphes suspensus*	JN623355
			Coruna clavata	JN623453
			Pachyneuron formosum	JN623464

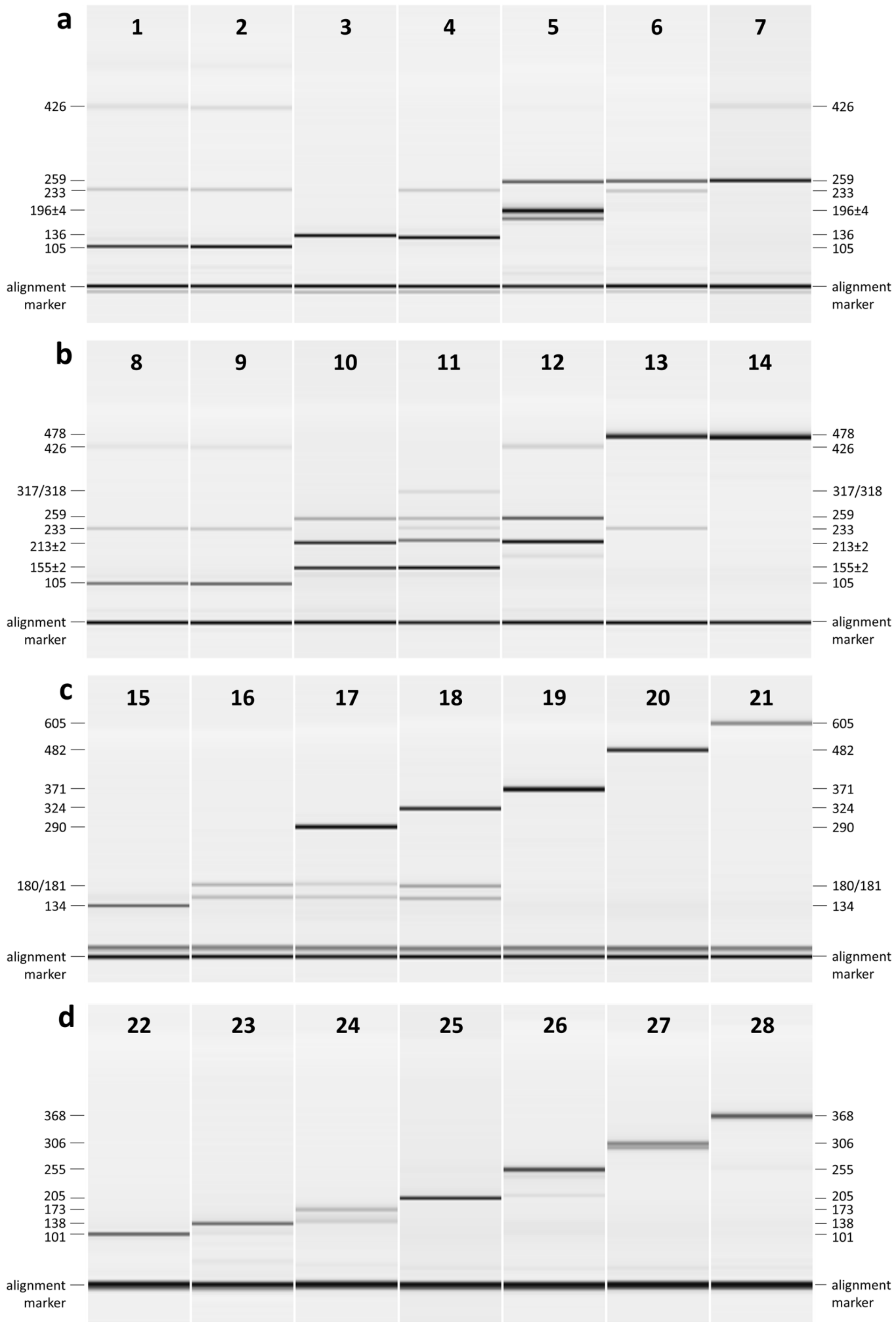

Figure S1: Qiaxcel gel view of amplicons generated by the diagnostic multiplex PCR assays from the field sample tests. The leftmost and rightmost numbers shows the amplicon lengths in base pairs. a)

Amplicons generated by 18SMP using aphid samples: *Metopolophium dirhodum* (1-2), *Rhopalosiphum padi* (3-4) and *Sitobion avenae* (5-7). b) Amplicons generated by 16SMP using aphid samples: *Metopolophium dirhodum* (8-9), *Sitobion avenae* (10-12) and *Rhopalosiphum padi* (13-14). c) Amplicons generated by PriMP using the aphid samples scored positive of Aphidiinae in 16SMP. Samples tested positive with *Ephedrus plagiator* (15), *Aphidius* spp. (16), *Aphidius uzbekistanicus* (17), *Aphidius ervi* (18), *Praon volucre* (19), *Praon gallicum* (20) and *Aphidius avenae* (21). d) Amplicons generated by HypMP using the aphid samples scored positive of either *Aphelinus*-hyperparasitoid or *Dendrocerus* in 16SMP. Samples tested positive *Alloxysta victrix* (22), *Asaphes suspensus* (23), *Asaphes vulgaris* (24), *Dendrocerus carpenteri* (25), *Coruna clavata* (26), *Phaenoglyphis villosa* (27) and *Aphelinus abdominalis* (28).

References

1 Fergusson, N. D. M. in *Handbooks for the Identification of British Insects* Vol. Vol. 8, Part 1c (eds P. C. Barnard & R. R. Askew) 1-55 (Royal Entomological Society of London, 1986).

2 Fergusson, N. D. M. A revision of the British species of *Dendrocerus* Ratzeburg (Hymenoptera: Ceraphronoidea) with a review of their biology as aphid hyperparasites. *Bulletin of the British Museum of Natural History (Entomology)* **41**, 255-314 (1980).

3 Gibson, G. A. P. The Australian species of *Pachyneuron* Walker (Hymenoptera: Chalcidoidea: Pteromalidae). *Journal of Hymenoptera Research* **10**, 29-54 (2001).

4 Gibson, G. A. P. & Vikberg, V. The species of *Asaphes* Walker from America north of Mexico, with remarks on extralimital distributions and taxa (Hymenoptera: Chalcidoidea, Pteromalidae). *Journal of Hymenoptera Research* **7**, 209-256 (1998).

5 Kamijo, K. & Takada, H. Aphid hyperparasites of the Pteromalidae occurring in Japan (Hymenoptera). in Studies on aphid hyperparasites of Japan, New Series 2. *Insecta Matsumurana*, 39-76 (1973).

6 Kavallieratos, N. G. *et al. Praon* Haliday (Hymenoptera : Braconidae : Aphidiinae) of southeastern Europe: key, host range and phylogenetic relationships. *Zoologischer Anzeiger* **243**, 181-209, doi:10.1016/j.jcz.2004.11.001 (2005).

7 Pennacchio, F. The Italian species of the genus *Aphidius* Nees (Hymenoptera, Braconidae, Aphidiinae). *Bollettino del Laboratorio di Entomologia Agraria Filippo Silvestri* **46**, 75-106 (1990).

8 Starý, P. Biosystematic synopsis of parasitoids on cereal aphids in the Western Palaearctic (Hymenoptera, Aphidiidae, Homoptera, Aphidoidea). *Acta Entomologica Bohemoslovaca* **78**, 382-396 (1981).

9 Starý, P. *Aphid parasites of Czechoslovakia. A review of the Czechoslovak Aphidiidae (Hymenoptera).* (Dr. W. Junk, 1966).

10 Graham, M. W. R. d. V. The Pteromalidae of north-western Europe (Hymenoptera: Chalcidoidea). *Bulletin of the British Museum of Natural History (Entomology)* **Suppl. 16** (1969).

11 Medvedev, G. S. (ed G. S. Medvedev) i-xii, 1-1341 (E.J. Brill, 1988).

12 Powell, W. The identification of hymenopterous parasitoids attacking cereal aphids in britain. *Systematic Entomology* **7**, 465-473, doi:10.1111/j.1365-3113.1982.tb00457.x (1982).

13 Tomanovic, Z. *et al.* Aphidius Nees aphid parasitoids (Hymenoptera, Braconidae, Aphidiinae) in Serbia and Montenegro: tritrophic associations and key. *Acta Entomologica Serbica* **8**, 15-39 (2003).

14 Takada, H. Parasitoids (Hymenoptera : Braconidae, Aphidiinae; Aphelinidae) of four principal pest aphids (Homoptera : Aphididae) on greenhouse vegetable crops in Japan. *Applied Entomology and Zoology* **37**, 237-249 (2002).

15 Japoshvili, G. & Abrantes, I. Aphelinus species (Hymenoptera : Aphelinidae) from the Iberian Peninsula, with the description of one new species from Portugal. *Journal of Natural History* **40**, 855-862, doi:10.1080/00222930600790737 (2006).

16 Luan, Y. X. *et al.* Ribosomal DNA gene and phylogenetic relationships of Diplura and lower Hexapods. *Science in China Series C-Life Sciences* **46**, 67-76, doi:10.1360/03yc9008 (2003).

17 Simon, C. *et al.* Evolution, weighting, and phylogenetic utility of mitochondrial gene-sequences and a compilation of conserved polymerase chain-reaction primers. *Annals of the Entomological Society of America* **87**, 651-701 (1994).

18 Kambhampati, S., Volkl, W. & Mackauer, M. Phylogenetic relationships among genera of Aphidiinae (Hymenoptera : Braconidae) based on DNA sequence of the mitochondrial 16S rRNA gene. *Systematic Entomology* **25**, 437-445, doi:10.1046/j.1365-3113.2000.00129.x (2000).

19 Kambhampati, S. & Smith, P. T. PCR primers for the amplification of four insect mitochondrial gene fragments. *Insect Molecular Biology* **4**, 233-236, doi:10.1111/j.1365-2583.1995.tb00028.x (1995).

20 Simon, C., Buckley, T. R., Frati, F., Stewart, J. B. & Beckenbach, A. T. Incorporating molecular evolution into phylogenetic analysis, and a new compilation of conserved polymerase chain reaction primers for animal mitochondrial DNA. *Annual Review of Ecology Evolution and Systematics* **37**, 545-579, doi:10.1146/annurev.ecolsys.37.091305.110018 (2006).

21 Folmer, O., Black, M., Hoeh, W., Lutz, R. & Vrijenhoek, R. DNA primers for amplification of mitochondrial cytochrome c oxidase subunit I from diverse metazoan invertebrates. *Molecular Marine Biology and Biotechnology* **3**, 294-299 (1994).

22 Sint, D., Raso, L. & Traugott, M. Advances in multiplex PCR: balancing primer efficiencies and improving detection success. *Methods in Ecology and Evolution* **3**, 898-905, doi:10.1111/j.2041-210X.2012.00215.x (2012).

23 Sandström, J. P., Russell, J. A., White, J. P. & Moran, N. A. Independent origins and horizontal transfer of bacterial symbionts of aphids. *Molecular Ecology* **10**, 217-228, doi:10.1046/j.1365-294X.2001.01189.x (2001).

24 Russell, J. A. & Moran, N. A. Horizontal transfer of bacterial symbionts: Heritability and fitness effects in a novel aphid host. *Applied and Environmental Microbiology* **71**, 7987-7994, doi:10.1128/aem.71.12.7987-7994.2005 (2005).

25 Traugott, M. & Symondson, W. O. C. Molecular analysis of predation on parasitized hosts. *Bulletin of Entomological Research* **98**, 223-231, doi:10.1017/s0007485308005968 (2008).

26 Ferrari, J., West, J. A., Via, S. & Godfray, H. C. J. Population genetic structure and secondary symbionts in host-associated populations of the pea aphid complex. *Evolution* **66**, 375-390, doi:10.1111/j.1558-5646.2011.01436.x (2012).
